# Demographic and clinical profiles of drug-induced psychosis: age, gender, and social context in a German cohort

**DOI:** 10.1007/s00406-026-02232-y

**Published:** 2026-03-31

**Authors:** Nadia Böss, Gabriele Koller, Kristina Adorjan, Bernhard Haller, Florian Eyer, Stefanie Geith

**Affiliations:** 1 Division of Clinical Toxicology and Poison Centre Munich, School of Medicine and Health, TUM University Hospital, Technical University of Munich, Ismaninger Str. 22, 81675 Munich, Germany; 2 Institute of AI and Informatics in Medicine, TUM School of Medicine and Health, Technical University of Munich, Ismaninger Str. 22, 81675 Munich, Germany; 3 Department of Psychiatry and Psychotherapy, LMU University Hospital, LMU Munich, Nußbaumstr. 7, 80336 Munich, Germany; 4 Institute of Psychiatric Phenomics and Genomics (IPPG), LMU University Hospital, LMU Munich, Nußbaumstr. 7, 80336 Munich, Germany; 5 Department of Psychiatry and Psychotherapy (UPD), University of Bern, Bolligenstrasse 111, 3000 Bern 60, Switzerland

**Keywords:** Drug-induced psychosis, Substance use disorder, Polysubstance use, Age differences, Gender differences, Social determinants, Psychotic symptoms

## Abstract

**Background:**

Drug-induced psychosis (DIP) represents a complex clinical challenge, particularly among younger substance-using populations. Recent epidemiological data indicate increasing DIP prevalence and decreasing age at first presentation, underscoring its growing clinical and public health relevance. While DIP arises in the context of substance use, distinguishing transient episodes from emerging primary psychotic disorders remains difficult, and data on demographic and clinical variations are limited.

**Methods:**

We conducted a descriptive cohort study of 340 patients diagnosed with DIP (ICD-10: F1X.5) between 2010 and 2020 at two university hospitals in Germany. We collected data from all eligible cases (n = 170) from a toxicology department (Technical University of Munich, TUM), and from the same number of cases from a psychiatry department (Ludwig-Maximilians-Universität in Munich, LMU). Variables assessed included sociodemographic data, psychiatric symptoms, substance use (self-report and toxicology), comorbidities, and family history. Analyses included descriptive statistics, chi-square or Fisher’s exact tests, and Mann–Whitney U tests to examine group differences, and Cohen’s Kappa coefficients to quantify agreement between self-reported and toxicologically confirmed substance use.

**Results:**

The cohort was predominantly male (77.4%) with a median age of 27 years. Migration background was present in 27.6%, with admissions peaking in 2015–2018. Psychotic symptoms were dominated by perceptual disturbances (62.3%) and thought disorder (76.3%). Women exhibited higher rates of tactile hallucinations, suicidal ideation, and depression (all p < 0.05) and tended to have higher serum ethanol levels. Daily substance use was common (69.0%), with polysubstance use exceeding 75%. Cannabis and stimulants predominated in younger patients, while opiates, sedatives, and ethanol were more frequent in older groups. Self-reported substance use showed fair to good agreement with toxicological findings, with the highest concordance for opiates and lower agreement for sedatives and ethanol.

**Conclusion:**

DIP showed clear gender- and age-related differences in psychotic symptom profiles and clinical presentation, alongside high rates of daily and polysubstance use, young male predominance, and migration-related patterns. These findings underscore the need for early, individualized, and demographically informed interventions.

**Supplementary Information:**

The online version contains supplementary material available at https://doi.org/10.1007/s00406-026-02232-y.

## Introduction

Drug-induced psychosis (DIP) refers to acute psychotic episodes triggered by psychoactive substances. Symptoms often resemble early manifestations of primary psychotic disorders, complicating differential diagnosis. Traditionally seen as a transient reaction to intoxication or withdrawal, DIP is now considered to signal an underlying vulnerability to chronic psychoses [1–3], underscoring the need for early detection and prevention, particularly in young adults.

Diagnosis is challenging because substance use disorders are common in schizophrenia [1], and symptom overlap with primary psychoses is substantial, especially in the acute phase [3, 4]. Longitudinal observation is often required to clarify whether symptoms remit with abstinence or progress to chronic psychosis [5]. Frequent polysubstance use, especially cannabis, stimulants, tramadol, and ethanol, further complicates causal attribution [6–9].

Cannabis is the best-studied DIP trigger, linked to higher incidence, more severe symptoms, and elevated risk of transition to schizophrenia spectrum disorders, particularly with early and regular use. However, this strong association may partly reflect the widespread use and comparatively high prevalence of cannabis relative to other DIP-associated substances [3, 4]. Methamphetamine shows a strong dose–response relationship, while opioids, sedatives, and novel psychoactive substances (NPS) add diagnostic complexity.

Recent epidemiological data indicate that the prevalence of DIP is increasing and that the age at first presentation is decreasing, particularly in Northern Europe between 2000 and 2016 [10]. These trends underline DIP’s growing clinical and public health significance and further justify the study’s focus on sociodemographic and clinical correlates in affected populations.

Age and gender shape DIP risk and presentation. Young men are most vulnerable, while women more often present with affective symptoms [10–12], highlighting the need for age- and gender-sensitive approaches.

Given these complexities and DIP’s potential to indicate underlying vulnerability, this study addresses four questions:

1. What are the sociodemographic characteristics of patients with DIP in a German cohort?
2. Which clinical symptoms and comorbidities occur, and how do these vary by age and gender?
3. What substance use patterns, including polysubstance use, are observed, and how do they differ by gender and age?
4. How do migration background and social factors influence DIP?

## Methods

### Study design and setting

We conducted a retrospective cohort study using data from the Department of Clinical Toxicology (Technical University of Munich, TUM) and the Department of Psychiatry (Ludwig Maximilian University of Munich, LMU), including admissions between August 2010 and August 2020.

**Fig. 1:**
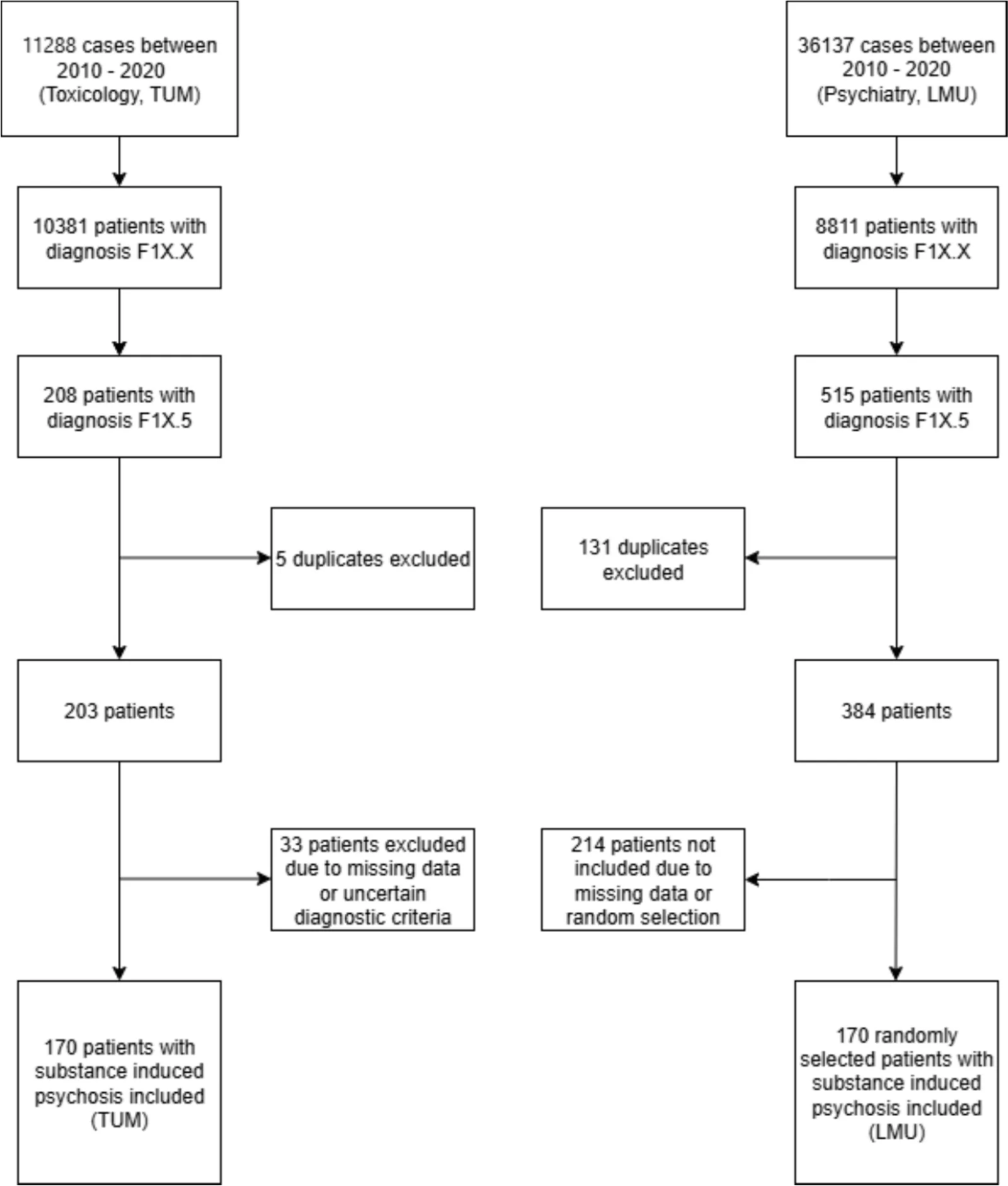
Flowchart of patient selection

### Participants

A total of 340 patients were included in the study, comprising 170 cases per institution. At TUM, all available cases meeting inclusion criteria were included. At LMU, cases were randomly selected from a predefined pool of patients diagnosed with DIP to match the sample size of the TUM cohort (Fig. 1). Random selection was performed by drawing physical patient records from the eligible pool. For patients with multiple admissions, only the first eligible case was included to avoid duplication.

Inclusion criteria were a diagnosis of drug-induced psychotic disorder (ICD-10: F1X.5), admission for psychiatric or toxicological treatment, recent substance use confirmed by self-report and toxicology (urine or serum), and psychotic symptoms consistent with DSM-5. All patients were clinically evaluated by a board-certified psychiatrist, who established the diagnosis of DIP based on reported symptoms, substance use pattern, and available clinical history.

Exclusion criteria were age under 16 years (treated in pediatric settings), absence of verified substance use or psychotic symptoms, and cases where schizophrenia spectrum disorder was judged more likely. Age was stratified into three groups (16–25, 26–40, 41–60 years) to reflect developmental stages and allow subgroup analyses.

The study was approved by the LMU Ethics Committee (No. 20/773, 27 Aug 2020) and conducted in accordance with the Declaration of Helsinki. Patient consent was waived due to the retrospective design.

### Data collection

Clinical data were extracted manually from patient records using a standardized protocol. Variables included sociodemographic, clinical, and substance-related information, as well as psychopathological symptoms across several domains. Toxicological screening was performed at both centers using immunoassays and confirmatory methods. Detailed assay descriptions are provided in the Supplementary Material.

### Statistical analysis

All statistical analyses were performed using IBM SPSS Statistics, Version 29. Descriptive statistics are presented as median (range) for quantitative data (mean ± SD for serum ethanol levels) and as absolute and relative frequencies for categorical variables. Group comparisons by sex and age group were conducted using Pearson’s chi-square tests or Fisher-based tests (Fisher’s exact or Fisher–Freeman–Halton, as appropriate) for categorical variables, and Mann–Whitney U tests for continuous non-normally distributed variables. Agreement between self-reported substance use and toxicological findings was quantified using Cohen’s Kappa coefficient for each substance class, based on dichotomous indicators of use. Gender differences in mean serum ethanol levels were tested using Welch’s t-test, with effect sizes reported as Cohen’s d.

Given the large number of comparisons in this exploratory study, p‑values are interpreted descriptively. To reduce the risk of false-positive findings, a more conservative threshold of 1% was used to denote statistically robust associations, whereas results with 1% *≤ * p < 5% are reported as nominal or trend-level findings.

Temporal variation in the proportion of patients with migration background was examined descriptively across admission periods (2010–2014, 2015–2018, 2019–2020).

## Results

### Descriptive analysis

#### Sociodemographic characteristics and migration background

**Fig. 2:**
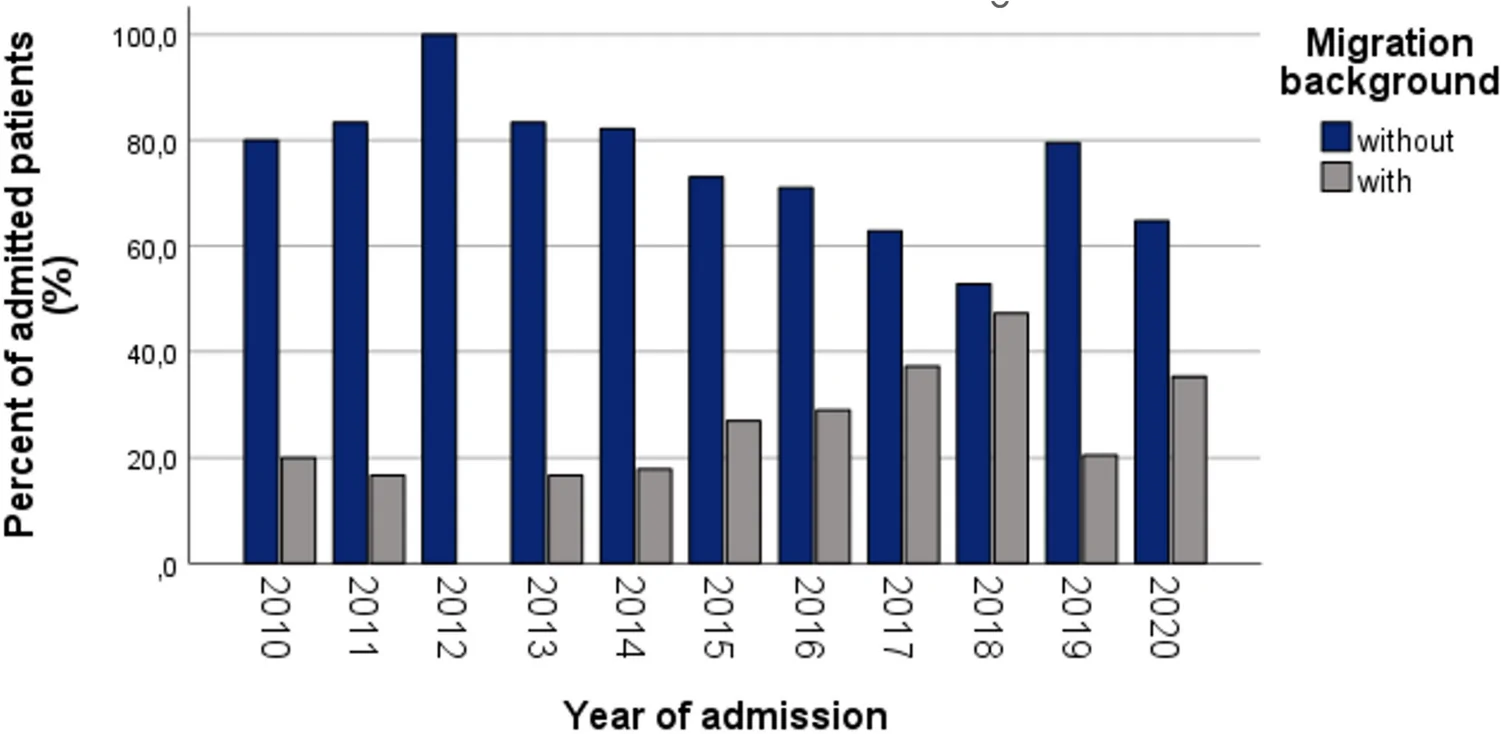
Percentage of patients with migration background. Data for 2012 included only patients without migration background

A total of 340 patients were included in the analysis. The median age was 27 years, with most participants being under the age of 40 (see Table 1). The majority were unmarried, while long-term partnerships and parenthood were uncommon. Educational and vocational levels were heterogeneous, with over half of the patients unemployed at the time of admission. Migration background was recorded in 27.6% overall and was more common among men. The proportion of patients with migration background was highest during 2015–2018 (see Fig. 2), coinciding with the peak in total admissions, and decreased slightly thereafter.

**Table 1:** Sociodemographic characteristics by gender and age

Characteristic	Total		Male		Female		p-value	Age						p-value
								*≤ * 25		26–40		*≥ * 41		
	n	(%)	n	(%)	n	(%)		n	(%)	n	(%)	n	(%)	
	340	(100)	263	(77.4)	77	(22.6)		145	(42.6)	155	(45.6)	40	(11.8)	
*Age*														
Median age (range)	27.0		27.0		27.0		0.842							
	(16–60)		(16-–59)		(17-–60)									
*Family situation*														
Unmarried	222	(92.1)	171	(91.4)	51	(94.4)	0.894	108	(97.3)	91	(87.5)	23	(88.5)	0.031*
Married	14	(5.8)	12	(6.4)	2	(3.7)		2	(1.8)	9	(8.7)	3	(11.5)	
Divorced	5	(2.1)	4	(2.1)	1	(1.9)		1	(0.9)	4	(3.8)	0	(0.0)	
Missing	99		76		23			34		51		14		
*Permanent relationship*														
Yes	58	(26.6)	43	(25.6)	15	(30.0)	0.536	17	(18.3)	28	(28.0)	13	(52.0)	**0**.**003**
No	160	(73.4)	125	(74.4)	35	(70.0)		76	(81.7)	72	(72.0)	12	(48.0)	
Missing	122		95		27			52		55		15		
*Migration background*														
Without	246	(72.4)	183	(69.6)	63	(81.8)	0.035*	108	(74.5)	106	(68.4)	32	(80.0)	0.257
With	94	(27.6)	80	(30.4)	14	(18.2)		37	(25.5)	49	(31.6)	8	(20.0)	
Missing	0		0		0			0		0		0		
*Parenthood*														
Yes	32	(16.8)	21	(14.6)	11	(23.9)	0.141	7	(8.4)	19	(22.4)	6	(27.3)	0.014*
No	158	(83.2)	123	(85.4)	35	(76.1)		76	(91.6)	66	(77.6)	16	(72.7)	
Missing	150		119		31			62		70		18		
*Education*														
Lower secondary	69	(34.7)	55	(35.5)	35	(53.8)	0.696	32	(30.8)	23	(30.7)	14	(70.0)	0.086
Intermediate secondary	45	(22.6)	35	(22.6)	10	(15.4)		23	(22.1)	20	(26.7)	2	(10.0)	
Higher secondary	49	(24.6)	39	(25.2)	10	(15.4)		25	(24.0)	21	(28.0)	3	(15.0)	
Vocational qualification	9	(4.5)	6	(3.9)	3	(4.6)		4	(3.8)	5	(6.7)	0	(0.0)	
School ongoing	8	(4.0)	7	(4.5)	1	(1.5)		7	(6.7)	1	(1.3)	0	(0.0)	
Other	3	(1.5)	3	(1.9)	0	(0.0)		2	(1.9)	1	(1.3)	0	(0.0)	
No certificate	16	(8.0)	10	(6.5)	6	(9.2)		11	(10.6)	4	(5.3)	1	(5.0)	
Missing	141		108		12			41		80		20		
*Qualification level*														
In training	47	(23.2)	39	(25.0)	8	(17.0)	0.552	38	(35.8)	9	(11.7)	0	(0.0)	< **0**.**001**
No qualification	63	(31.0)	44	(28.2)	19	(40.4)		41	(38.7)	18	(23.4)	4	(20.0)	
Skilled worker	50	(24.6)	39	(25.0)	11	(23.4)		12	(11.3)	28	(36.4)	10	(50.0)	
Commercial school	25	(12.3)	21	(13.5)	4	(8.5)		13	(12.3)	8	(10.4)	4	(20.0)	
College	5	(2.5)	4	(2.6)	1	(2.1)		1	(0.9)	4	(5.2)	0	(0.0)	
University	13	(6.4)	9	(5.8)	4	(8.5)		1	(0.9)	10	(13.0)	2	(10.0)	
Missing	137		107		30			39		78		20		
*Currently working*														
Yes	85	(37.9)	67	(38.5)	18	(36.0)	0.748	37	(37.4)	38	(39.2)	10	(35.7)	0.935
No	139	(62.1)	107	(61.5)	32	(64.0)		62	(62.6)	59	(60.8)	18	(64.3)	
Missing	116		89		27			46		58		12		

p-values based on Pearson’s $\chi ^2$ test; Fisher’s exact test applied for variables with expected cell counts < 5; Mann–Whitney U test used for continuous variables (age)

p-values are interpreted descriptively; values *< 0.01* (in bold) are considered statistically robust, and values between 0.01 and 0.05 (marked with *) indicate nominal (trend-level) findings

### Anamnestic data

Psychiatric comorbidities were common among the study population (see Table 2). Substance use disorders were the most frequently documented additional diagnoses, followed by depression and borderline personality disorder (BPD), with smaller numbers presenting with conditions such as panic disorder, post-traumatic stress disorder (PTSD), or attention deficit hyperactivity disorder (ADHD).

Regarding familial predisposition, a high proportion of patients (75.5%) reported a family history of psychiatric illness, including substance use disorders, affective disorders, and schizophrenia-spectrum disorders (see Table 5).

**Table 2:** Psychiatric comorbidities by gender and age group

	Total (positive)		Missing	Male		Female		p-value	Age						p-value
Diagnosis									*≤ * 25		26–40		*≥ * 41		
	n	(%)		n	(%)	n	(%)		n	(%)	n	(%)	n	(%)	
Psychiatric comorbidity	255	(75.2)	1	200	(76.3)	55	(71.4)	0.372	106	(73.6)	115	(74.2)	34	(85.0)	0.310
Substance use disorder	196	(57.8)	1	152	(58.0)	44	(57.1)	0.892	79	(54.9)	87	(56.1)	30	(75.0)	0.063
Depression	39	(11.6)	3	25	(9.6)	14	(18.2)	0.044*	18	(12.5)	16	(10.5)	5	(12.5)	0.835
Panic disorder	7	(2.1)	2	6	(2.3)	1	(1.3)	1.000	3	(2.1)	4	(2.6)	0	(0.0)	0.876
PTSD$^{\text {a}}$	9	(2.7)	2	6	(2.3)	3	(3.9)	0.431	4	(2.8)	5	(3.2)	0	(0.0)	0.799
BPD$^{\text {b}}$	15	(4.4)	2	9	(3.4)	6	(7.8)	0.118	8	(5.6)	7	(4.5)	0	(0.0)	0.366
ADHD$^{\text {c}}$	21	(6.2)	2	16	(6.1)	5	(6.5)	1.000	13	(9.0)	8	(5.2)	0	(0.0)	0.094

p-values based on Pearson’s $\chi ^2$ test; Fisher’s exact test applied for variables with expected cell counts <5

p-values *<0.01* are considered statistically robust, and values between 0.01 and 0.05 (marked with *) indicate nominal (trend-level) findings

$^{\text {a}}$Posttraumatic Stress Disorder, $^{\text {b}}$Borderline Personality Disorder, $^{\text {c}}$Attention Deficit Hyperactivity Disorder

#### Substance use patterns

The median age at first substance use was 15 years (range 8–57). Most participants (69.0%) reported daily use, while roughly one-quarter used only a single substance and others up to five or more (Table 3).

Cannabis, stimulants, and ethanol were most frequently reported, whereas NPS were used by approximately one quarter of the sample. Smaller proportions reported opiates, sedatives, or hallucinogens.

Toxicological analyses revealed THC as the most frequently detected substance, followed by sedatives—primarily benzodiazepines—and stimulants. Opiates were identified in about one-eighth of cases, while ethanol and NPS were less common. Complete results of all substances detected in the toxicological analysis can be found in the Supplementary Material.

**Table 3:** Substance use patterns by gender and age group

Characteristic	Total		Male		Female		p-value	Age						p-value
								*≤ * 25		26–40		*≥ * 41		
	n	(%)	n	(%)	n	(%)		n	(%)	n	(%)	n	(%)	
	340	(100)	263	(77.4)	77	(22.6)		145	(42.6)	155	(45.6)	40	(11.8)	
*Age at first consumption*														
Median age (range)	15.0	(8–57)	15.0	(12–57)	14.5	(8–27)	0.506							
*Consumption pattern*														
Daily	225	(69.0)	178	(70.6)	47	(63.5)	0.338	91	(65.5)	103	(91.2)	31	(79.5)	0.176
Weekly	27	(8.3)	18	(7.1)	9	(12.2)		17	(12.2)	9	(8.0)	1	(2.6)	
Irregular	74	(22.7)	56	(22.2)	18	(24.3)		31	(22.3)	1	(0.9)	7	(17.9)	
Missing	14		11		3			6		42		1		
*Number of used substances*														
1 Substance	79	(23.6)	58	(22.3)	21	(28.0)	0.251	33	(22.9)	37	(24.0)	9	(24.3)	0.430
2 Substances	116	(34.6)	92	(35.4)	24	(32.0)		47	(32.6)	57	(37.0)	12	(32.4)	
3 Substances	82	(24.5)	65	(25.0)	17	(22.7)		43	(29.9)	32	(20.8)	7	(18.9)	
4 Substances	40	(11.9)	28	(10.8)	12	(16.0)		13	(9.0)	19	(12.3)	8	(21.6)	
*≥ *5 Substances	18	(5.4)	17	(6.5)	1	(1.3)		8	(5.6)	9	(5.8)	1	(2.7)	
Missing	5		3		2			1		1		3		
*Self-reported substance use*														
Ethanol	181	(53.2)	141	(53.6)	40	(51.9)	0.797	74	(51.0)	82	(52.9)	25	(62.5)	0.432
Sedatives$^{\text {a}}$	52	(15.3)	40	(15.2)	12	(16.0)	0.867	12	(8.3)	31	(20.1)	9	(22.5)	**0**.**006**
Opiates$^{\text {b}}$	65	(19.1)	48	(18.3)	17	(22.4)	0.422	17	(11.7)	33	(21.4)	15	(37.5)	**0**.**001**
Stimulants$^{\text {c}}$	178	(52.4)	144	(54.8)	34	(44.7)	0.124	82	(56.6)	81	(52.6)	15	(37.5)	0.102
Hallucinogens$^{\text {d}}$	35	(10.3)	30	(11.4)	5	(6.6)	0.287	20	(13.8)	14	(9.1)	1	(2.5)	0.090
THC	214	(62.9)	165	(62.7)	49	(65.3)	0.681	109	(75.7)	89	(57.8)	16	(40.0)	**0**.**001**
NPS$^{\text {e}}$	84	(24.7)	68	(26.0)	16	(21.3)	0.415	35	(24.3)	39	(25.5)	10	(25.0)	0.984
*Toxicological analysis*														
Ethanol$^{\text {f}}$	25	(7.4)	15	(5.7)	10	(13.0)	0.031*	7	(4.8)	12	(7.7)	6	(15.0)	0.272
Sedatives$^{\text {a}}$	118	(34.7)	94	(35.7)	24	(31.2)	0.391	47	(32.4)	55	(35.5)	16	(40.0)	0.269
Benzodiazepines	108	(31.8)	87	(33.1)	21	(27.3)	0.236	47	(32.4)	48	(31.0)	13	(32.5)	0.729
Opiates$^{\text {b}}$	44	(12.9)	29	(11.0)	15	(19.5)	0.077	7	(4.8)	24	(15.5)	13	(32.5)	**<0.001**
Stimulants$^{\text {c}}$	80	(23.5)	63	(24.0)	17	(22.0)	0.754	27	(18.6)	42	(27.1)	11	(27.5)	0.116
Cocaine	34	(10.0)	24	(9.1)	10	(13.0)	0.385	10	(6.9)	18	(11.6)	6	(15.0)	0.144
Amphetamines	51	(15.0)	41	(15.6)	10	(13.0)	0.585	20	(13.8)	27	(17.4)	4	(10.0)	0.563
THC	131	(38.5)	100	(38.0)	31	(40.3)	1.000	63	(43.4)	58	(37.4)	10	(25.0)	0.160
NPS$^{\mathrm{e}}$	18	(6.8)	13	(5.0)	5	(6.5)	0.538	5	(3.4)	12	(7.7)	1	(2.5)	0.399

p-values based on Pearson’s $\chi ^2$ test; Fisher’s exact test (or Fisher–Freeman–Halton for RxC tables) applied when expected cell counts <5. Mann–Whitney U test used for continuous variables (age)

p-values *<0.01* (in bold) are considered statistically robust, and values between 0.01 and 0.05 (marked with *) indicate nominal (trend-level) findings

$^{\text {a}}$ Sedatives include benzodiazepines, GHB/GBL, pregabalin, and ketamine

$^{\text {b}}$Opiates include Opiates, Buprenorphin, Fentanyl, EDDP

$^{\text {c}}$Stimulants: includes Cocaine, Amphetamines, Methamphetamine, Methylphenidate

$^{\text {d}}$Hallucinogens: inludes LSD and Psylocybin

$^{\text {e}}$Novel Psychoactive Substances: includes synthethic Cannabinoids and Cathinones

$^{\text {f}}$cut-off 0.2 g/L in serum

#### Concordance between self-reported and toxicologically confirmed substance use

Concordance analysis was performed for six substance classes with available paired self-report and toxicological data (Table 4). Cohen’s Kappa values ranged from 0.304 to 0.747, all statistically significant (p < 0.001). Opiates demonstrated the highest concordance, followed by THC and NPS. Sedatives showed the lowest concordance.

Underreporting was low for opiates, stimulants, THC, ethanol, and NPS, but reached 28.6% for sedatives. Overreporting ranged from 2.5% (sedatives) to 29.2% (ethanol). Self-report sensitivity was above 87% for all substances except sedatives (32.2%). Specificity was highest for sedatives (95.7%) and opiates (92.8%).

Hallucinogens were excluded from the concordance analysis, because toxicological screening was limited to LSD only.

**Table 4:** Concordance between self-reported and toxicologically confirmed substance use

Substance class	N valid	Concordant		Both positive		Overreport		Underreport		Sensitivity	Specificity	Kappa	p-value
		n	(%)	n	(%)	n	(%)	n	(%)	%	%		
Opiates$^{\text {a}}$	279	258	(92.5)	40	(14.3)	17	(6.1)	4	(1.4)	90.9	92.8	0.747	0.001
Stimulants$^{\text {b}}$	278	191	(68.7)	70	(25.2)	77	(27.7)	10	(3.6)	87.5	61.1	0.389	0.001
THC	286	215	(75.2)	120	(42.0)	60	(21.0)	11	(3.8)	91.6	61.3	0.514	0.001
Ethanol$^{\text {c}}$	113	78	(69.0)	23	(20.4)	33	(29.2)	2	(1.8)	92.0	62.5	0.377	0.001
Sedatives$^{\text {d}}$	280	193	(68.9)	38	(13.6)	7	(2.5)	80	(28.6)	32.2	95.7	0.304	0.001
NPS$^{\text {e}}$	115	90	(78.3)	16	(13.9)	23	(20.0)	2	(1.7)	88.9	76.3	0.442	0.001

*Sensitivity* corresponds to the proportion of positive toxicology tests coherent with patient self-report

*Specificity* corresponds to the proportion of negative toxicology tests coherent with patient self-report

Kappa: < 0.20 poor, 0.21–0.40 fair, 0.41–0.60 moderate, 0.61–0.80 good, > 0.80 excellent

$^{\text {a}}$Opiates: opiates, buprenorphine, fentanyl, EDDP

$^{\text {b}}$Stimulants: cocaine, amphetamines, methamphetamine and MDMA

$^{\text {c}}$Ethanol: cut-off 0.2 g/L in serum

$^{\text {d}}$Sedatives: benzodiazepines, GHB/GBL, pregabalin, and ketamine

$^{\text {e}}$NPS: Synthetic cannabinoids and cathinons

#### Psychiatric symptoms

The most frequently documented clinical symptoms included perceptual disturbances, thought disorder, and restlessness (see Table 5). Hallucinations were common, particularly auditory and visual types, while tactile hallucinations were less frequently observed. Disturbances of self-awareness and affective impairment were also noted in a considerable number of cases, alongside widespread reports of sleep disruption.

Regarding risk behaviors, nearly one-third of patients exhibited signs of self-harm risk, with smaller proportions presenting a risk to others or reporting suicidal ideation.

**Table 5:** Family history and symptom profiles by gender and age group

Characteristic	Total		Missing	Male		Female		p-value	Age						p-value
									*≤ 25*		26–40		*≥ 41*		
	n	(%)	n	n	(%)	n	(%)		n	(%)	n	(%)	n	(%)	
*Family history*															
Substance abuse disorder	39	(20.2)	147	27	(18.2)	12	(26.7)	0.288	21	(20.6)	15	(20.5)	3	(16.7)	0.932
Schizophrenia, schizotypal or delusional disorders	28	(14.6)	148	25	(17.0)	3	(6.7)	0.096	16	(15.7)	9	(12.5)	3	(16.7)	0.826
Affective disorder	56	(29.2)	148	39	(26.5)	17	(37.8)	0.189	36	(35.3)	16	(22.2)	4	(22.2)	0.146
Neurotic, stress-related or somatoform disorders	6	(3.1)	148	4	(2.7)	2	(4.4)	0.626	4	(3.9)	1	(1.4)	1	(5.6)	0.463
Personality or behavioural disorders	3	(1.6)	148	0	(0.0)	3	(6.7)	0.012*	2	(2.0)	0	(0.0)	1	(5.6)	0.194
Behavioural abnormalities	7	(3.6)	148	6	(4.1)	1	(2.2)	1.000	4	(3.9)	2	(2.8)	1	(5.6)	0.727
Behavioural or emotional disorders (childhood onset)	2	(1.0)	148	2	(1.4)	0	(0.0)	1.000	1	(1.0)	1	(1.4)	0	(0.0)	1.000
Suicide$^{\text {a}}$	10	(5.2)	148	9	(6.1)	1	(2.2)	0.457	5	(4.9)	4	(5.6)	1	(5.6)	1.000
*Symptoms*															
Perceptual disturbance	210	(62.3)	3	159	(61.2)	51	(66.2)	0.503	92	(63.9)	92	(59.7)	26	(66.7)	0.637
Visual hallucination	106	(31.5)	3	81	(31.2)	25	(32.5)	0.827	45	(31.2)	45	(29.2)	16	(41.0)	0.365
Tactile hallucination	32	(9.5)	3	20	(7.7)	12	(15.6)	0.038*	10	(6.9)	16	(10.4)	6	(15.4)	0.246
Olfactory hallucination	2	(0.6)	3	1	(0.4)	1	(1.3)	0.405	1	(0.7)	1	(0.6)	0	(0.0)	1.000
Auditory hallucination	135	(40.1)	3	97	(37.3)	38	(49.4)	0.058	61	(42.4)	57	(37.0)	17	(43.6)	0.573
Attention or memory impairment	147	(43.9)	5	110	(42.5)	37	(48.7)	0.337	68	(47.6)	61	(39.9)	18	(46.2)	0.394
Disorders of thought content or form	257	(76.3)	3	193	(74.2)	64	(83.1)	0.128	116	(80.6)	111	(72.5)	30	(75.0)	0.264
Thought disorder	177	(52.8)	5	135	(52.3)	42	(54.5)	0.732	85	(59.0)	73	(48.0)	19	(48.7)	0.143
Paranoid delusion	163	(48.5)	4	124	(47.9)	39	(50.6)	0.669	63	(43.8)	78	(51.3)	22	(55.0)	0.292
Delusional thinking	142	(42.1)	3	108	(41.5)	34	(44.2)	0.683	59	(41.0)	64	(41.8)	19	(47.5)	0.757
Affective impairment	178	(53.1)	5	132	(51.2)	46	(59.7)	0.186	85	(59.0)	76	(50.0)	17	(43.6)	0.133
Impairment of drive or motivation	273	(81.0)	3	212	(81.5)	61	(79.2)	0.624	115	(79.9)	125	(81.7)	33	(82.5)	0.892
Lack of drive	134	(40.0)	5	101	(39.1)	33	(42.9)	0.560	63	(43.8)	61	(40.1)	10	(25.6)	0.123
Decline in performance	72	(21.5)	5	53	(20.5)	19	(24.7)	0.438	29	(20.1)	30	(19.7)	13	(33.3)	0.159
Restlessness	229	(67.9)	3	180	(69.2)	49	(63.6)	0.355	91	(63.2)	106	(69.3)	32	(80.0)	0.117
Sleep disturbance	173	(51.5)	4	135	(52.1)	38	(49.4)	0.669	78	(54.2)	70	(45.8)	25	(64.1)	0.086
Disturbance of self-awareness	112	(33.5)	6	86	(33.5)	26	(33.8)	0.961	62	(43.1)	41	(27.0)	9	(23.7)	**0**.**005**
Anxiety or compulsivity-related symptoms	166	(49.3)	3	124	(47.7)	42	(54.5)	0.302	66	(45.8)	78	(51.0)	22	(55.0)	0.500
Anxiety	164	(48.7)	3	122	(46.9)	42	(54.5)	0.240	65	(45.1)	77	(50.3)	22	(55.0)	0.466
Obsessive-compulsive symptoms	7	(2.1)	4	6	(2.3)	1	(1.3)	0.697	3	(2.1)	2	(1.3)	2	(5.0)	0.281
Harm potential	123	(36.3)	1	88	(33.6)	35	(45.5)	0.060	53	(36.6)	56	(36.4)	14	(35.0)	0.983
Risk of self-harm	95	(28.0)	1	69	(26.3)	26	(33.8)	0.202	41	(28.3)	42	(27.3)	12	(30.0)	0.939
Risk of harm to others	60	(17.8)	2	49	(18.8)	11	(14.3)	0.365	22	(15.2)	31	(20.3)	7	(17.5)	0.516
Suicidal ideation	48	(14.4)	6	31	(12.1)	17	(22.1)	0.028*	21	(14.6)	21	(13.9)	6	(15.4)	0.968

p-values based on Pearson’s $\chi ^2$ test; Fisher’s exact test (or Fisher–Freeman–Halton for RxC tables) applied when expected cell counts < 5

p-values *<0.01* (in bold) are considered statistically robust, and values between 0.01 and 0.05 (marked with *) indicate nominal (trend-level) findings

$^{\text {a}}$ Family history of suicide

### Gender-specific differences

Age distribution was similar between men and women, with identical median ages (Table 1). While most participants in both groups were unmarried, women more frequently reported a permanent relationship or parenthood, though these differences were not statistically significant.

Men tended to more frequently have a migration background (30.4% vs. 18.2%, p = 0.035*). Educational and employment status showed no significant differences, though women more often reported only lower secondary education or no vocational qualification, while men were more often undergoing training.

Women tended to more often present with detectable serum ethanol (38.5% vs. 17.2%; p = 0.031*) and tended to show higher mean serum ethanol levels at admission (0.80 ± 1.22 g/L) than in men (0.40 ± 0.94 g/L), although this difference did not reach statistical significance (Welch’s t(34.38) = –1.55, *p = 0.13*, Cohen’s *d = 0.40*). Although no significant gender differences emerged regarding age at first use, consumption patterns, or the number of substances used, certain trends were apparent: women more often reported single-substance use, whereas men more frequently reported stimulant and hallucinogen use.

Clinical profiles were largely comparable, but women tended to more often report tactile hallucinations (15.6% vs. 7.7%, p = 0.038*) and suicidal ideation (22.1% vs. 12.1%, p = 0.028*) (Table 5). Family history was similar across groups, with a trend toward more frequent personality or behavioral disorders being reported only by women (6.7% vs. 0.0%, p = 0.012*).

### Age group-specific differences

Distinct sociodemographic and substance use patterns emerged across age groups (Tables 1, 3). Younger patients (*≤ 25* years) showed a trend toward more often being single (p = 0.031*), while older patients more frequently reported a stable relationship or parenthood. Vocational training was most common among the youngest and employment rates were similar across groups.

Age at first use did not differ, but daily consumption peaked in the 26–40 group. Older patients more often reported multiple substances, though not significantly. THC detection declined with age, whereas opiates and sedatives were more common in older participants.

Clinically, disturbances of self-awareness were significantly more frequent in the youngest group, while no further robust age-related differences appeared in psychopathology or family history (Table 5).

## Discussion

This study offers a descriptive analysis of a large clinical DIP sample from psychiatric and toxicological settings. A major strength is the integration of self-reported and toxicologically verified substance data, including serum ethanol levels, enabling a more nuanced characterization of use patterns than studies relying solely on retrospective reports.

Most patients were young men (median age 27), consistent with epidemiological data showing that DIP predominates in this group [2, 8, 10, 13–16]. Scandinavian registry studies report 75–80% male cases with median ages around 30, similar to UK cohorts [10, 14]. Young men are also at elevated risk of progression to schizophrenia-spectrum disorders [2, 17], and recent data suggest decreasing age of onset, particularly for cannabis- and stimulant-related cases [10], underscoring the need for early prevention.

Low educational attainment and unemployment in our cohort align with known social determinants of psychosis [18], and similar associations between socioeconomic adversity and polysubstance use have been reported internationally [19, 20].

The peak in migration background during 2015–2018 coincides with the European refugee crisis [21] and reflects evidence of increased psychosis risk among migrants and refugees [22–24]. The proportion of the German population with migration background rose from 19.2% in 2010[25] to 26.7% in 2020 [26] , with the steepest increases between 2015 and 2018 [27, 28]. Our cohort’s overall prevalence of 27.6% is comparable to the general population in 2019–2020. This pattern is consistent with socio-developmental models linking migration-related adversity and structural disadvantage to increased psychosis risk [29]. The need for culturally sensitive, trauma-informed care in urban settings remains critical [30–33].

Polysubstance use was common, with cannabis, stimulants, and ethanol most frequent, consistent with international findings [6, 19, 20, 34]. Age gradients were evident: THC use was highest in patients *≤ * 25 and declined with age, whereas sedatives and opiates increased among older groups. These patterns parallel international data [9] and general population trends, where benzodiazepine prescribing peaks in midlife while misuse is more common in young adults [35]. Taken together, our results suggest that sedative and opiate use in DIP largely reflects prescribing and long-term use, whereas cannabis remains more accessible in youth.

Self-reported substance use showed fair to good agreement with toxicological findings, with the highest concordance observed for opiates and lower concordance for sedative substances. Overreporting emerged as the predominant discordance pattern for most substances (stimulants, THC, ethanol, NPS), while sedatives such as benzodiazepines and pregabalin were characterized by marked underreporting despite high specificity of negative self-reports, consistent with their frequent therapeutic use in the acute setting [36]. Despite the long detection window of THC, agreement between self-report and toxicology remained only moderate, which is in line with findings that intermittent consumption patterns and the timing of sampling substantially limit assay sensitivity. Novel psychoactive substances showed only moderate agreement between self-report and analytical detection, reflecting both limited coverage of specialized testing panels and patients’ incomplete knowledge of the exact substances taken, so that self-report can provide important complementary information beyond targeted toxicological screening [37].

Gender differences in substance use were less pronounced. Although the difference did not reach statistical significance, women more often tested positive for ethanol and showed higher mean serum levels (0.8 g/L vs. 0.4 g/L), consistent with sex-specific pharmacokinetics and neurobiology [38–42]. These factors plausibly explain the observed trend.

Clinical presentation in our cohort was dominated by positive psychotic symptoms, particularly perceptual disturbances, thought disorder, and restlessness, consistent with prior descriptions of acute DIP [1, 43, 44]. Younger patients more frequently exhibited disturbances of self-awareness, aligning with previous evidence of limited insight and ongoing neurocognitive maturation in early psychosis [45]. These features highlight the typically acute, affectively charged, and fluctuating nature of DIP presentations, which may complicate initial diagnostic differentiation and require developmentally tailored treatment approaches.

Women differed from men mainly in affective and somatic symptoms, with indications of higher rates of tactile hallucinations, suicidal ideation, and depression. These findings support the notion of an affective subtype of DIP in women, characterized by greater affective burden and suicidality [46–51]. Elevated ethanol levels may further increase suicide risk, consistent with evidence that alcohol is a strong predictor of suicidal behavior in females [52]. Longitudinal data suggest that women with DIP are more likely to progress to bipolar disorder, whereas men more often convert to schizophrenia-spectrum disorders [11, 12]. Notably, only women reported a family history of personality or behavioral disorders, which may indicate gender-specific familial transmission pathways [53, 54]. However, as multiple comparisons were performed, these differences should be interpreted with caution.

Comorbidity rates were high, particularly for substance use disorders, consistent with diagnostic overlap and shared vulnerability in DIP populations [9]. Beyond depression, no other comorbidities differed by gender. Women showed a trend toward higher rates of depression, reflecting the higher prevalence of depressive disorders in women [55–57], likely due to hormonal, psychosocial, and trauma-related factors [47, 49, 50]. These patterns support the view that DIP in women may represent an affective subtype, while men’s risk is more closely linked to psychotic trajectories. Compared to first-episode psychosis, where men more often meet criteria for substance use disorders [48], this suggests divergent exposure pathways.

Our study has several methodological strengths, including a large sample size, dual-center design, and the integration of toxicological, clinical, and demographic data. However, its retrospective design required reliance on clinical documentation and incomplete toxicological testing, particularly for substances with short detection windows such as GHB, LSD, and psilocybin, which may have led to missed exposures and greater dependence on self-report, with associated recall bias and underreporting. Toxicological testing was not uniformly available across substances, with ethanol, NPS, and hallucinogens assessed only in subsamples, potentially introducing selection bias and limiting generalizability for these classes. Differences between toxicological detection windows and the broader clinical timeframe covered by self-report likely contributed to apparent over- or underreporting, particularly for THC, where prolonged metabolite detectability does not necessarily correspond to recent use. For sedatives, the inability to distinguish recreational from therapeutic administration in the emergency setting further complicates interpretation of discordant cases. Finally, the use of dichotomous indicators of use precluded analyses of dose, frequency, and temporal proximity to symptom onset, which may be relevant for understanding the clinical impact of different substance use patterns. Diagnostic assessment was based on clinical ICD-10 judgments without standardized instruments, potentially reducing reliability, although all cases were evaluated by board-certified psychiatrists. Migration background was recorded as a binary variable without information on timing, origin, or legal status, preventing analysis of migration-specific risk factors such as recency of arrival, trauma exposure, or acculturation stress. The temporal fluctuations observed in our cohort may reflect changing migration flows, but should be interpreted cautiously given these data limitations. The imbalanced gender distribution (77% male) limits power for female-specific analyses. Finally, the absence of follow-up prevents evaluation of diagnostic stability or long-term trajectories, in line with previous studies reporting low diagnostic stability of DIP and frequent conversion to primary psychotic disorders [3, 5, 58, 59]. Additionally, data were collected from two German hospitals in urban settings, which may limit generalizability to rural areas, other regions, or healthcare systems with different referral patterns and treatment models.

Clinically, our findings highlight the need for individualized care that considers developmental stage, gender, and migration-related vulnerabilities. The high prevalence of polysubstance use and diagnostic uncertainty, particularly with NPS, call for broader toxicological screening, longitudinal follow-up, and closer psychiatry–toxicology collaboration.

## Supplementary Information

Below is the link to the electronic supplementary material.Supplementary file 1 (pdf 118 KB)

## Data Availability

No datasets were generated or analysed during the current study.
