# Supplementary material for: Demographic and clinical profiles of drug-induced psychosis: age, gender, and social context in a German cohort
Source: Eur Arch Psychiatry Clin Neurosci. 2026 Mar 31;276(6):2937–51. doi: 10.1007/s00406-026-02232-y (PMC13525961; doi:10.1007/s00406-026-02232-y)
Supplement: Supplementary file 1 [file 406_2026_2232_MOESM1_ESM.pdf]

Supplementary Material

Substance classification

Self-reported and toxicologically confirmed substances were categorized into pharmacologically meaningful groups to facilitate analysis. The following classification scheme was applied:

- **Stimulants:** amphetamine, methamphetamine, cocaine, and MDMA.
- **Opioids:** heroin, morphine, methadone, fentanyl, tramadol, and other opiate derivatives.
- **Sedatives:** benzodiazepines, GHB/GBL, pregabalin, and ketamine.
- **New psychoactive substances (NPS):** synthetic cathinones and synthetic cannabinoids.
- **THC**
- **Hallucinogens:** LSD and psilocybin-containing mushrooms.
- **Ethanol**

Toxicological analyses

At TUM, urine analyses were conducted for all patients using an AU480 chemistry analyzer (Beckman Coulter, Brea, CA, USA) [1] with an immunoassay test panel. The panel included amphetamines/MDMA, benzoylecgonine (cocaine metabolite), buprenorphine, barbiturates, benzodiazepines, delta-9-THC, ethanol, ethyl glucuronide, methadone metabolite (2-ethylidene-1,5-dimethyl-3,3-diphenylpyrrolidine), fentanyl, GHB, opiates, pregabalin, and synthetic cannabinoids (Spice-1, Spice-2, Spice-3). Dipstick tests were additionally used for selected substances such as LSD, ketamine, or tilidine. Between 2014 and mid-2020, online solid-phase extraction (SPE) high-performance liquid chromatography with diode array detection (HPLC-DAD; TOX.I.S. II, Shimadzu, Kyoto, Japan) was applied to identify substances not covered by the immunoassay and to allow for differentiation. This method enabled the detection of oxycodone, tilidine, tramadol, zolpidem, zopiclone, certain cathinones/phenethylamines, and ketamine.

At LMU, urine drug screening was performed using immunoassays. Benzodiazepines, amphetamines, cocaine, opiates, methadone, buprenorphine, and THC were analyzed with the CEDIA Drug Screening Assay (Thermo Fisher Scientific, Waltham, MA, USA). Fentanyl was measured using the ARK Fentanyl Assay (Specialty Diagnostix GmbH, Bad Homburg, Germany). All assays were carried out on the Indiko Plus analyzer (Thermo Fisher Scientific, Waltham, MA, USA) [2].

Complete Toxicological Analysis Results

Table 1: Substance use patterns by gender and age group (excerpt)

**Characteristic**	Total		Male		Female		p-value	Age						p-value
								$\leq$25		26-40		$\geq$41		
	n	(%)	n	(%)	n	(%)		n	(%)	n	(%)	n	(%)	
	340	(100)	263	(77.4)	77	(22.6)		145	(42.6)	155	(45.6)	40	(11.8)	
**Toxicological analysis**														
Ethanol[1]	25	(7.4)	15	(5.7)	10	(13.0)	0.031*	7	(4.8)	12	(7.7)	6	(15.0)	0.272
Sedatives[2]	118	(34.7)	94	(35.7)	24	(31.2)	0.391	47	(32.4)	55	(35.5)	16	(40.0)	0.269
Benzodiazepines	108	(31.8)	87	(33.1)	21	(27.3)	0.236	47	(32.4)	48	(31.0)	13	(32.5)	0.729
Pregabalin	17	(5.0)	15	(5.7)	2	(2.6)	0.241	1	(0.7)	10	(6.5)	6	(15.0)	**0.001**
Ketamin	1	(0.3)	1	(3.8)	0	(0.0)	1.000	0	(0.0)	1	(0.6)	0	(0.0)	0.474
GHB/GBL	5	(1.5)	4	(1.5)	1	(1.3)	1.000	1	(0.7)	3	(1.9)	1	(2.5)	0.690
Opiates (all[3])	44	(12.9)	29	(11.0)	15	(19.5)	0.077	7	(4.8)	24	(15.5)	13	(32.5)	**<0.001**
Opiates	18	(5.3)	16	(6.1)	2	(2.6)	0.262	0	(0.0)	10	(6.5)	8	(20.0)	**0.001**
Buprenorphin	17	(5.0)	10	(3.8)	7	(9.1)	0.103	4	(2.8)	7	(4.5)	6	(15.0)	**0.010**
Fentanyl	7	(2.1)	4	(1.5)	3	(3.9)	0.359	2	(1.4)	4	(2.6)	1	(2.5)	0.726
EDDP	12	(3.5)	5	(1.9)	7	(9.1)	**0.008**	2	(1.4)	7	(4.5)	3	(7.5)	0.066
Stimulants[4]	80	(23.5)	63	(24.0)	17	(22.0)	0.754	27	(18.6)	42	(27.1)	11	(27.5)	0.116
Cocaine	34	(10.0)	24	(9.1)	10	(13.0)	0.385	10	(6.9)	18	(11.6)	6	(15.0)	0.144
Amphetamines	51	(15.0)	41	(15.6)	10	(13.0)	0.585	20	(13.8)	27	(17.4)	4	(10.0)	0.563
Methamphetamine	7	(2.1)	7	(2.7)	0	(0.0)	0.330	1	(0.7)	5	(3.2)	1	(2.5)	0.307
Methylphenidate	3	(0.9)	2	(0.8)	1	(1.3)	0.476	1	(0.7)	1	(0.6)	1	(2.5)	0.525
MDMA	9	(2.6)	7	(2.7)	2	(2.6)	1.000	3	(2.1)	4	(2.6)	2	(5.0)	0.791
LSD	4	(1.2)	3	(1.1)	1	(1.3)	1.000	3	(2.1)	1	(0.6)	0	(0.0)	0.790
THC	131	(38.5)	100	(38.0)	31	(40.3)	1.000	63	(43.4)	58	(37.4)	10	(25.0)	0.160
NPS[5]	18	(6.8)	13	(5.0)	5	(6.5)	0.538	5	(3.4)	12	(7.7)	1	(2.5)	0.399

[1]cut-off 0.2 g/L in serum[2]Sedatives include benzodiazepines, GHB/GBL, pregabalin, and ketamine [3]Opiates include Opiates, Buprenorphin, Fentanyl, EDDP

[4]Stimulants: includes Cocaine, Amphetamines, Methamphetamine, Methylphenidate

[5]Novel Psychoactive Substances: includes synthethic Cannabinoids and Cathinones

p-values based on Pearson's χ^2 test; Fisher's exact test (or Fisher–Freeman–Halton for RxC tables) applied when expected cell counts <5. Mann–Whitney U test used for continuous variables (age).

p-values < 0.01 are considered statistically robust, and values between 0.01 and 0.05 (marked with *) indicate nominal (trend-level) findings.

References

[1] Beckman Coulter, Inc.: AU480 Chemistry Analyzer. Accessed: 2025-09-02 (2025). https://www.beckmancoulter.com/de/products/chemistry/au480

[2] Thermo Fisher Scientific Inc.: Indiko Plus Clinical Chemistry Analyzer. Accessed: 2025-09-02 (2025). https://www.thermofisher.com/order/catalog/product/98640000
